# A systematic review and meta-analysis of the normal reference value of the longitudinal left atrial strain by three dimensional speckle tracking echocardiography

**DOI:** 10.1038/s41598-022-08379-7

**Published:** 2022-03-15

**Authors:** Reza Mohseni-Badalabadi, Tayebeh Mirjalili, Arash Jalali, Tahereh Davarpasand, Ali Hosseinsabet

**Affiliations:** 1 Cardiology Department, Tehran Heart Center, Tehran University of Medical Sciences, Tehran, Iran; 2 Research Department, Tehran Heart Center, Tehran University of Medical Sciences, Tehran, Iran

**Keywords:** Cardiology, Medical research

## Abstract

The normal reference value of the global longitudinal left atrial strain during the reservoir phase (LASr) by 3D speckle-tracking echocardiography (3DSTE) is needed to define the abnormal and normal spectra and to compare and interpret the obtained values. The present study is a meta-analysis of 3DSTE-derived normal reference value of the longitudinal LASr and an attempt to determine probable contributing factors in the variations of reported ranges. The databases of PubMed, Scopus, and Embase were searched for the following keywordS: “Left atrial/left atrium” and “strain/speckle/deformation” and "three-dimensional/3-dimensional/three dimensional/3 dimensional/three dimension/3 dimension/three-dimension/3-dimension/3D/3-D". The studies selected included those on adult healthy subjects without cardiovascular risk factors. A random-effect model was used to calculate the global 3DSTE-derived longitudinal LASr, and meta-regression was applied to determine inter-study heterogeneity. Our search yielded 316 adult subjects from 5 studies. The mean value of the global 3DSTE-derived longitudinal LASr was 27.5% (95% CI, 25.2–29.8%). There was significant heterogeneity between the studies. The meta-regression analysis revealed the publication year, the heart rate, and systolic and diastolic blood pressure as the sources of heterogeneity. The current meta-analysis determined a normal reference value of the global 3DSTE-derived longitudinal LASr of 27.5% (95% CI, 25.2–29.8%). The heterogeneity between studies may be explained by the publication year, the heart rate, and systolic and diastolic blood pressure.

## Introduction

The left atrium (LA) is a chamber between the pulmonary veins and the left ventricle (LV). The LA wall is thin by comparison with the LV wall. The chamber regulates LV filling and works in interaction with the LV. The LA has 3 phasic functions during the cardiac cycle: reservoir, whereby blood is stored during the LV systole; conduit, whereby blood is transferred to the LV in early diastole; and contraction, whereby blood is pushed into the LV in late diastole^1^.

The LA phasic functions can be evaluated by several modalities such as echocardiography, computed tomography, and cardiac magnetic resonance imaging^2^. The phasic functions of the LA can also be evaluated by several echocardiographic methods such as 2D or 3D volumetric parameters, pulsed-wave Doppler, tissue Doppler imaging, and myocardial deformation imaging^3^. Not only are the deformation markers less load-dependent^2^ and less affected by tethering motion, but also they are angle independent^4^. The use of 2D speckle-tracking echocardiography (2DSTE) has been widespread for the evaluation of the LA phasic functions, and the modality has received attention from echocardiography societies, which have presented standardization recommendations^5^. However, 3D deformation markers have been validated in experimental and human studies^6^, and deformational parameters obtained by 3D speckle-tracking echocardiography (3DSTE) have more agreement with cardiac magnetic resonance tagging than those obtained by 2DSTE^7^. By comparison with 2DSTE, 3DSTE is not affected by out-of-plane motion or twisting motion produced by motion in the third direction^8^. Moreover, 3DSTE is a feasible and reproducible method^6,8^ that has been applied to evaluate the LA phasic functions in various clinical conditions such as atrial fibrillation, hypertrophic cardiomyopathy, hypertension, and healthy subjects^9–13^.

Whereas 2DSTE-derived normal values for the LA deformation indices have already been presented^14^, 3DSTE-derived normal reference values for the LA deformation indices have yet to be determined. Normal reference values are necessary for the recognition of abnormal values, and they can lead to more clinical application of the LA assessment by 3DSTE.

The measurement of the longitudinal LA strain during the reservoir phase by 3DSTE (3DLASr) or 2DSTE (2DLASr) has been done in most published articles in this field rather than strain values in other directions or phases^15^. Accordingly, in the present study, we sought to determine not only normal reference values for the longitudinal 3DLASr in adults through a systematic review and a meta-analysis but also the cause of variations in values.

## Methods

### Search profile

The conduct of the present study was in keeping with Preferred Reporting Items for Systematic Reviews and Meta-Analyses (PRISMA) guidelines^16^. The databases of PubMed, Scopus, and Embase were searched for the following keywords: “Left atrial/left atrium" and “strain/speckle/deformation” and "three-dimensional/3-dimensional/three dimensional/3 dimensional/three dimension/3 dimension/three-dimension/3-dimension/3D/3-D" (Supplement 1). Another strategy was to perform a reference search to identify related studies. The search was restricted to the English language. The search was done on April 29, 2021, and the study was registered at PROSPERO on March 13, 2021 (CRD42021236533).

### Study selection

Studies were included if they reported longitudinal LASr as numbers (the mean and the standard deviation vs only the mean in figures) in healthy subjects. Exclusion criteria consisted of animal studies, review articles, case reports, letters to editors, editorials, and conference abstracts. Also excluded were studies without control groups, studies with control groups containing healthy subjects aged below 18 years, studies featuring control group patients with cardiovascular risk factors, studies with inadequate descriptions of control groups regarding the absence of cardiovascular risk factors, and studies reporting the averaged values of the LA segments rather than the global value. This stage was done independently by 3 investigators (T.M., R.M.B., and A.H.). Disagreements were resolved according to consensus between A.H. and T.D.

### Data collection

The eligible articles were reviewed by the three investigators (T.M., R.M.B., and A.H.) independently. Study characteristics, clinical data, echocardiographic method characteristics, echocardiography data, and 3DLASRr were extracted. The disagreements were resolved according to consensus between A.H. and T.D. Among the studies that used the same data set, the studies selected were those that (a) included control subjects not matched with a case group, (b) were not confined to a special subgroup, and (c) had more control subjects.

### Statistical analysis

The statistical analysis was done by Stata software, version 16 (College Station, TX: StataCorp LLC). The mean and the 95% confidence interval (CI) of 3DLASr were computed by the random-effects model. The Cochrane’s Q test (*P* < 0.1) and the I^2^ statistic were used for the evaluation of heterogeneity between the studies. The results were illustrated as a forest plot. Clinical and demographic data, if reported by more than 2 studies, were considered a possible source of 3DLASr variations and evaluated by meta-regression to estimate their effects on the variation of the normal reference value of 3DLASr. The results were presented as β and 95% CI. The stability of the estimated normal reference value of 3DLASr was presented through a comparison between the random-effects model and a fixed-effects model by excluding a study conducted with General Electric software and by excluding the studies that had less CI overlap with other studies. Publication bias was assessed by the Egger test (*P* < 0.1).

The quality of the studies, including internal and external validity criteria, was assessed by the quality evaluation tool, presented by Downs and Black (1998)^17^. The tool has also been used by other researchers^14,18^. Similar to other meta-analyses in this field^14,18^, in the present study, criteria considered for study quality included inter and intraobserver variability, the blindness of the operators who obtained images and analyzed videos, the reporting of the heart rate and systolic and diastolic blood pressure, and the approach to the computation of 3DLASr. The quality of the selected studies was checked by T.M., R.M.B., and A.H. independently, and disagreements were resolved by consensus between A.H. and T.D.

## Results

### Study selection

**Figure 1:**
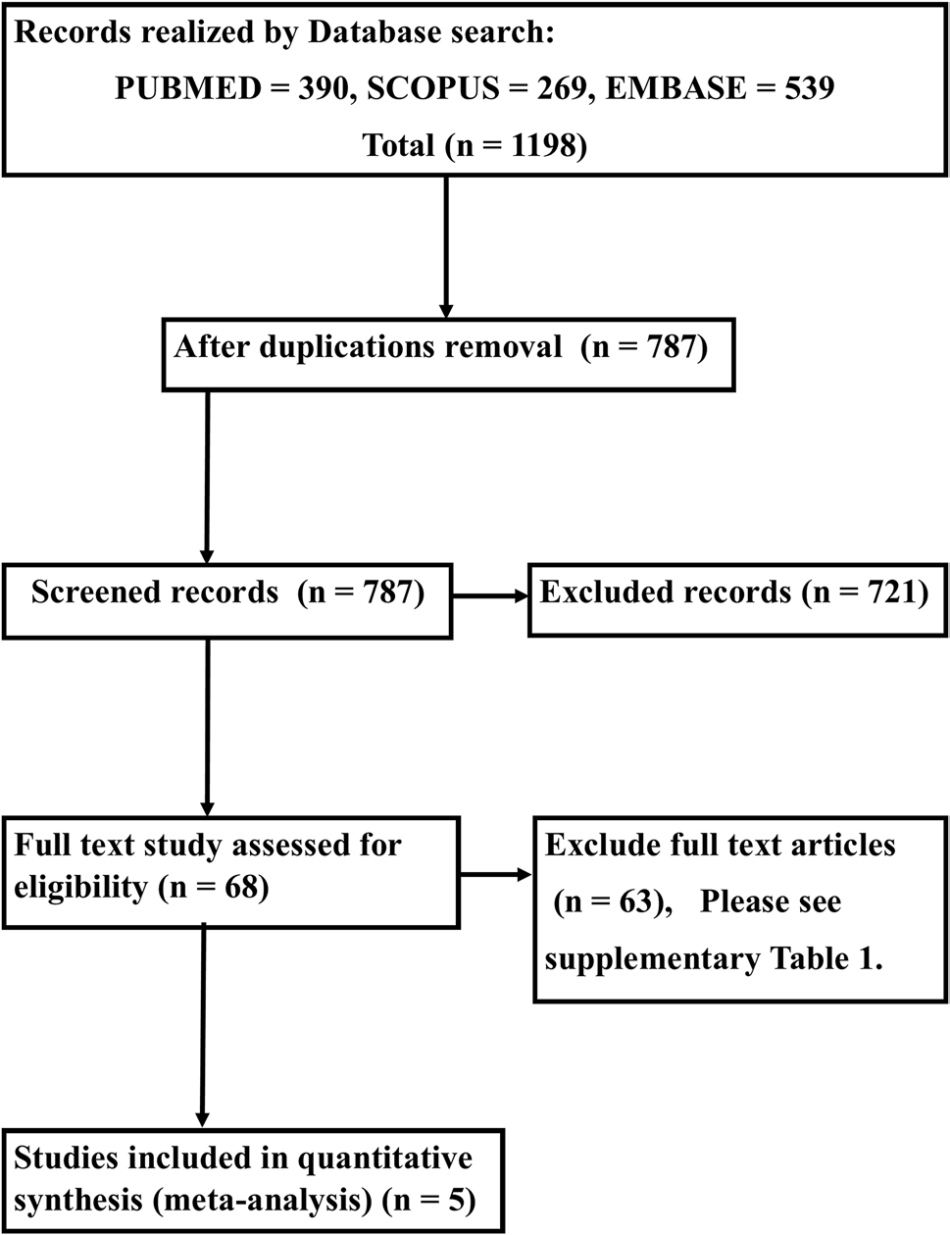
The image illustrates the study design and the preferred reporting items for systematic reviews and meta-analyses flowchart, presenting the selection process of studies. The reasons for full-text exclusion are demonstrated in Supplemental Table 1S.

Figure 1 demonstrates the PRISMA flowchart of this study. Our search procedure identified 1198 studies. After the exclusion of duplications, 787 articles were entered in the screening stage. The titles and abstracts of these studies were reviewed to identify the studies that fulfilled our inclusion and exclusion criteria. Sixty-eight studies were eligible for full-text review. Reference search was not added to other studies. Sixty-three studies were excluded based on the exclusion criteria (Supplemental Table 1S). Ultimately, 5 studies were included. The baseline data of these studies are presented in Table 1. These studies were conducted on a total of 316 subjects. Four studies were performed with Toshiba software and 1 study with General Electric software. The mean age range of the subjects was 32 to 54 years, and sex composition was 44–79% for males. The other presented data were compatible with healthy subjects. Two studies measured 2DLASr and 3DLASr concomitantly^9,13^.

**Table 1:** Study characteristics.

Study	Year	Country	N	Age (years)	Male (%)	HR (bpm)	BMI (kg/m^2^)	BSA (m^2^)	SBP (mmHg)	DBP (mmHg)	LVEF (%)	LAVI (ml/m^2^)	E/e′ ratio	VR (Hz)	Platform	Software	Probe	Gating	Model (segments)	Sub volume (N)	Disease/condition studied
Mochizuki et al.	2013	Japan	77	32.3 ± 14.2	62	67.6 ± 12.3	21.9 ± 2.7	NR	112.7 ± 10.2	72.5 ± 9.5	68.6 ± 4.6	21.7 ± 6.3	S: 5.4 ± 1.3 L: 4.6 ± 1.1	20 ± 1	Artida, Toshiba	Toshiba Medical Systems	PST-25SX	R–R	16	4	Atrial fibrillation
Aly et al.	2014	Netherland	29	46 ± 16	79	69 ± 11	NR	1.8 ± 0.12	NR	NR	59 ± 5	28 ± 7	S: 5.7 ± 1.5 L: 8.3 ± 1.6 A: 6.9 ± 1.4	23 ± 12	Artida, Toshiba	Toshiba Medical Systems	PST–25SX	R–R	16	4	Hypertrophic cardiomyopathy
Piros et al.	2016	Hungary	34	36.1 ± 11.2	44	NR	NR	NR	NR	NR	63.7 ± 8.2	NR	?6.21 ± 1.75	NR	Toshiba	3D Wall Motion Tracking, version 2.7	PST-25SX	R–R	NR	6	Healthy subjects for evaluation of Left atrial ejection force
Esposito et al.	2019	Italy	82	54.30 ± 11.17	59	60–80	23.8 ± 3.12	1.84 ± 0.19	116.41 ± 9.61	73.98 ± 7.72	60.36 ± 5.12	19.79 ± 6.46	L: 5.78 ± 1.44	NR	Toshiba	3D Wall Motion Tracking, version 2.5	PST-25SX	R–R	NR	6	Hypertension and paroxysmal atrial fibrillation
Nabeshima et al.	2021	Japan	94	44.3 ± 15.4	57	65 ± 10	NR	1.71 ± 0.18	129 ± 10	76 ± 9	55 ± 4	25.9 ± 6.3	A: 6.05 (5.11–7.36)	27 ± 4	GE	4D Auto LAQ	4 V or 4Vc	R–R	NR	NR	Healthy and patients for obtaining normal value references of left atrial strain

?, not defined.

*A* averaged septal and lateral value, *BMI* body mass index, *BSA* body surface area, *DBP* diastolic blood pressure, *HR* heart rate, *L* lateral, *LAVI* left atrial volume index, *LVEF* left ventricular ejection fraction, *NR* not reported, *S* septal, *SBP* systolic blood pressure, *VR* volume rate.

### Normal strain value

**Figure 2:**
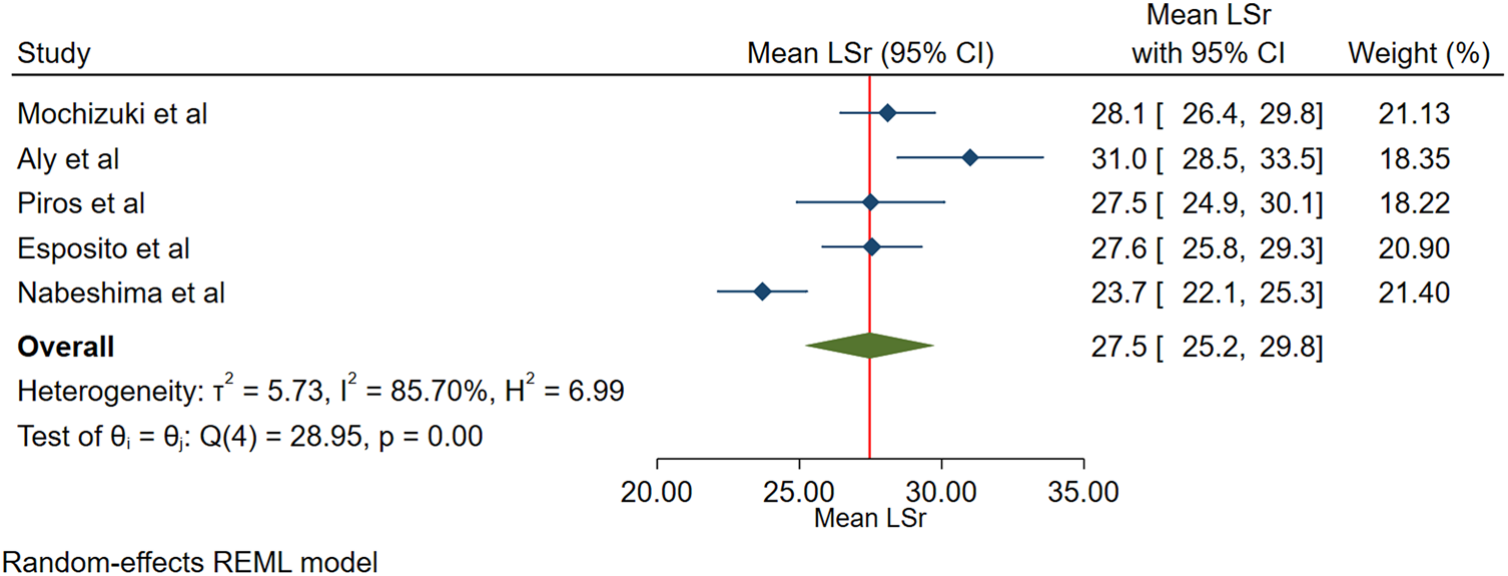
The image depicts the normal range of the longitudinal 3D speckle-tracking echocardiography-derived left atrial strain during the reservoir phase.

The pooled normal value for the global longitudinal 3DLASr was 27.5% (95% CI, 25.2–29.8%). The range of the global longitudinal 3DLASr was 23.7–31.0%. The heterogeneity between the studies was significant (Q = 29, *P* < 0.001, I^2^ = 85.7%) (Fig. 2).

**Figure 3:**
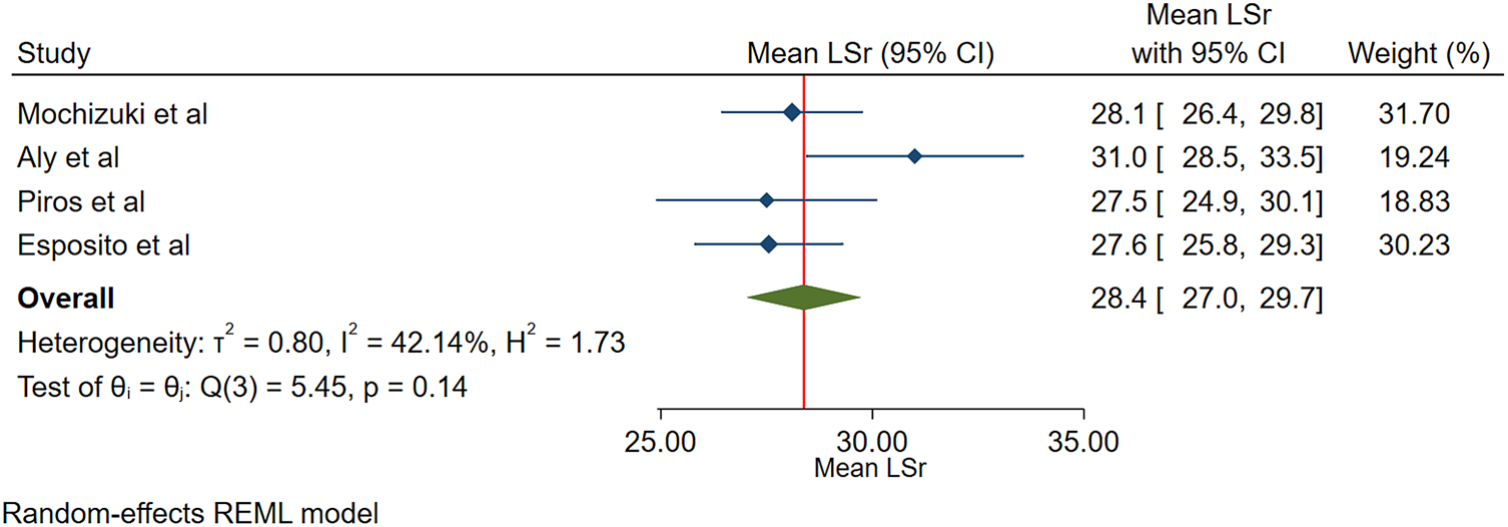
The image shows the normal range of the longitudinal 3D speckle-tracking echocardiography-derived left atrial strain during the reservoir phase analyzed by Toshiba software.

The sensitivity analysis with fixed effects models presented the mean value for the global longitudinal 3DLASr of 26.9% (95% CI, 26.1–27.8%). Following the exclusion of the General Electric software-based study, the mean value of the global longitudinal 3DLASr was 28.4% (95% CI, 27.0–29.7%) according to the random-effects model. The Toshiba-based study forest plot is presented in Fig. 3. Following the exclusion of 2 studies, one of which was the General Electric software-based study, on account of having less CI overlap with the other studies, the mean value for the global longitudinal 3DLASr was 27.8% (CI, 26.7–28.9%).

The origin of heterogeneity was evaluated by meta-regression. The publication year (β =  − 0.60, *P* = 0.026), the heart rate (β = 1.78, *P* ≤ 0.001), and systolic (β =  − 0.28, *P* ≤ 0.001) and diastolic (β =  − 1.30, *P* = 0.002) blood pressure were recognized as the sources of inter-study heterogeneity (Table 2).

**Table 2:** Meta-regression analysis for longitudinal three-dimensional speckle tracking echocardiography derived left atrial strain during reservoir phase.

Variables	Numbers of study	β (95% CI)	P-value
Year of publication	5	−0.60 (−1.12 to −0.07)	0.026
Number of participants	5	−0.07 (−0.13 to 0.00)	0.056
Race (Non-Asian vs Asian)	5	−2.75 (−7.11 to 1.62)	0.218
Age, years	5	−0.00 (−0.34 to 0.33)	0.978
Sex (male)	5	0.13 (−0.07 to 0.33)	0.202
Heart rate, bpm	3	1.78 (1.12 to 2.43)	< 0.001
Body surface area, m^2^	3	38.04 (−37.05 to 113.14)	0.321
Systolic blood pressure, mmHg	3	−0.28 (−0.41 to −0.15)	< 0.001
Diastolic blood pressure, mmHg	3	−1.30 (−2.11 to −0.49)	0.002
Left ventricular ejection fraction, %	5	0.21 (−0.30 to 0.71)	0.425
Maximal left atrial volume index, mL/m^2^	4	0.10 (−0.99 to 1.19)	0.856
Subvolumes	4	−0.84 (−2.25 to 0.56)	0.240
Volume rate, (Hz)	3	−0.69 (−2.19 to 0.81)	0.367

*CI* confidence interval.

The assessment of publication bias revealed no significant publication bias for the global longitudinal 3DLASr (*P* = 0.150 for the Egger test).

Two studies^9,13^ reported 2DLASr values of 35.8 ± 7.7% and 39.4 ± 12.1% and 3DLASr values of 28.1 ± 7.4% and 23.7 ± 7.6%, respectively. The mean difference value and the standard deviation of these two methods in these investigations were 7.7 ± 8.1% and 15.7 ± 12.1%, respectively, with a *P* value of less than 0.001 in both studies.

### Quality of studies

The included studies satisfied five to eight items of 11 items designed for quality evaluation. One study satisfied fewer than 50% of the items, two studies satisfied 55% of the items, and two studies satisfied about 70% of the items. All the studies described their objectives, outcomes, main findings, and strain imaging protocol. Intra- and interobserver variability was reported by four out of the five studies (Supplemental Table 2S).

## Discussion

We are the first to present the normal reference value of the global longitudinal 3DLASr through a meta-analysis. The LA works in interaction with the LV during the cardiac cycle. At the ventricular systole, blood enters the LA through the pulmonary veins while the LV contracts, which pulls the mitral annulus toward the LV apex to help the LA expand^15^. Thus, the LA deformation during the LV systole is dependent on the innate properties of the LA and LV function. Accordingly, LASr can reflect fibrosis in the LA myocardium^19^ and the properties of the LV such as the LV end-diastolic pressure^20^. Previous research has provided the clinical values of LASr in the prediction of clinical conditions such as exercise capacity^21^ and LV dysfunction grading^22^, as well as future cardiovascular events such as survival and the recurrence of atrial fibrillation^23,24^.

A major method for the evaluation of the LA function is 2DSTE^5^, with meta-analyses having already presented the normal reference value for its markers^14^. Still, this method has some weaknesses. A fixed plane is required for 2DSTE, which means that speckles during cardiac cycles should not exit the defined plane. The translational motion of the heart or myocardial motion in another direction precludes the satisfaction of this assumption. The evaluation of the global strain needs images from multiple planes during different cardiac cycles. Although 3DSTE provides images more rapidly than 2DSTE and does not require imaging in multiple planes and during a different cardiac cycle, it suffers from lower spatial and time resolution and stitch artifacts due to multi-beat acquisition^6,8,25^. There is a paucity of research on the comparison between 2 and 3DSTE for the prediction of clinical events, but it has been demonstrated that 3DSTE is stronger for the prediction of atrial fibrillation recurrence and the differentiation of patients with paroxysmal atrial fibrillation from the control group^9,26^.

Comparison of 2DLASr and 3DLASr in the two included studies revealed that 2DLASr was more than 3DLASr in both investigations, which is compatible with the findings of a previous meta-analysis^14^. The meta-analysis reported an approximate normal reference value for 2DLASr of 39.4% (95% CI, 38.0–40.8%), which is more than the value we obtained for 3DLASr (ie, 27.5%) (95% CI, 25.2–29.8%). The aforementioned comparisons between the 2 methods may explain the difference between these two values. It is worthy to cite that our meta-analysis on 3DLASr is on a lower scale than previous meta-analyses on 2DLASr.

### Demographic characteristics

We did not find age and sex as a source of heterogeneity, which chimes in with the finding of a previous meta-analysis regarding 2DLASr^14^. Although it does not mean that factors such as age exert no effects on the LA reservoir function, it does indicate that these factors are not a source of variability in the normal reference value presented in the included studies. A reduction in 2DLASr in tandem with increasing age has been demonstrated in several studies^27–31^, but we stress that the age range in the studies included in the present meta-analysis was not wide (32–54 years). A previous investigation demonstrated an increase in 3DLASr after 50 years^32^. In most of the studies included in our meta-analysis, the participants were younger than 50 years of age^30^.

Sex was not a determinant of 2DLASr in most studies subjected to meta-analysis^27–31^. In a study that evaluated 3DLASr in men and women, the results showed no difference between the two sexes vis-à-vis this marker^32^. Likewise, we did not find sex to be a source of inter-study heterogeneity.

Comparison of 2DLASr between European and Asian races in a previous investigation demonstrated no difference between the two ethnic groups^30^, which is concordant with our findings.

Whereas a meta-analysis on 2DLASr found a correlation between the body surface area and 2DLASr^14^, only three studies reported the body surface area with a very narrow range in our meta-analysis.

### Hemodynamic variables

The heart rate is the source of heterogeneity in studies regarding 2DLASr^14^. Nonetheless, several studies have demonstrated that the heart rate is not an independent determinant of 2DLASr^28,29,31^. Our results showed the heart rate as the source of inter-study heterogeneity. It is worthy of note that the range of the heart rate in the studies included in our meta-analysis was narrow (65–69 bpm), with only three studies having reported the heart rate as the mean and the standard deviation. It is, therefore, possible that the heart rate by chance presented itself as a source of inter-study heterogeneity.

Blood pressure in healthy subjects is associated with 2DLASr^29,31^. The findings of our study are compatible with the studies reporting this association.

### Echocardiography data

The LV systolic function is correlated with the LA reservoir function^28,33^. The LV ejection fraction is a marker of the LV systolic function, and 3DLASr is a marker of the LA reservoir function. In our study, the mean LV ejection fraction was within the normal range, so the LV ejection fraction was not a source of heterogeneity between the included studies. The LA volume index was correlated with 2DLASr in some studies^27,30,31^. The LA volume index in the included studies was lower than that in studies that confirmed the correlation between 2DLASr and the LA volume index^27,31^.

Subvolume numbers (4 vs 6) were not correlated with 3DLASr. The increased subvolume was accompanied by an increased frame rate as an index of temporal resolution, but it seems that it was not a determinant of heterogeneity between the included studies.

Our exclusion of the single General Electric software-based study resulted in no significant changes in the normal range of LASr.

We found the publication year to be a source of heterogeneity. It is possible that the time elapsed since the first 3DLASr measurement is allied to more expertise in this regard.

The finding of the source of heterogeneity in our study by meta-regression was affected by the low number of studies included. In addition, not all the included studies provided the details of the study population such as the body mass index or the body surface area, clinical data such as blood pressure, or echocardiography data such as the LA volume index, precluding an in-depth analysis. The factors may, therefore, be a source of heterogeneity between studies.

### Publication bias

The results of our meta-analysis showed no significant publication bias, although the limited number of included studies precluded exact results. It should be, however, noted that we searched studies via a hierarchical method whereby three independent researchers reviewed all abstracts and selected full texts; additionally, all discrepancies were resolved according to consensus between two of the researchers. This checking method lessened the probability of missing related manuscripts.

Our study results yielded the normal reference range of 3DLASr, although the range was obtained from a low number of suitable studies. Our findings can be used in clinical practice until future studies provide more robust data regarding 3DLASr among healthy normal subjects. These values may assist in the longitudinal assessment of patients who suffer from chronic conditions such as heart failure with reduced or preserved ejection fractions or those who were exposed to acute events such as myocardial infarction because they can depict changes from the normal range to the abnormal range.

Our study revealed a need for more studies regarding the normal reference value of 3DLASr because of the dearth of high-quality studies in this field. Indeed, the majority of the studies that were suitable for our meta-analysis did not measure deformation in other directions or during the conduit or contraction phase. In addition, data are absent regarding the comparison of 3DLASr and the value obtained by cardiac magnetic resonance imaging.

### Study limitations

The present meta-analysis incorporated five studies on a total of 316 subjects. This sample size is low by comparison with studies via 2DSTE. We are hopeful that the publication of more studies investigating deformation in other directions and in other phasic times will contribute to more optimal meta-analyses since we were not able to provide values for these markers in our study.

The studies included in our meta-analysis were observational or case–control, which are associated with high heterogeneity compared with randomized control trials because of the objective entity of these types of studies^34^.

The software that is used for 3DSTE is semiautomatic, and the competency of the operators who obtain deformation data is unknown. In our meta-analysis, we considered the same value for all the studies included. The absence of large-scale studies yielding preliminary normal reference values should be addressed in the future.

We could not conclusively assess the difference in vendors since we had four studies performed with Toshiba software and only one study conducted with General Electric software.

The fact that the study populations in the case and control groups of all the studies incorporated in the current meta-analysis were Asian also prevented us from demonstrating any possible differences concerning the normal reference value of 3DLASr between ethnicities.

Another weakness of note is that we analyzed study data, and not patient data, on account of the unavailability of the former. We also assessed the quality of the included studies according to the checklist used in similar meta-analyses in this field^14,35^. Although this checklist prevents significant bias in the evaluation of studies, it is not objective totally^36^. What can also be deemed a limitation is that we did not exclude any studies because of low quality. Still, the overall quality of the included studies, albeit not excellent, was by no means poor insofar as they satisfied about 60% of the required items on average.

Finally, we were not able to conduct a stratified meta-analysis by considering the quality of studies because of the innate properties of quality^37^.

## Conclusions

We demonstrated a mean global 3DSTE-derived longitudinal LASr of 27.5% (95% CI, 25.2–29.8%) in normal healthy subjects. The heterogeneity in different normal reference values was related to the publication year, the heart rate, and systolic and diastolic blood pressure. Further studies in this field are required to confirm the normal reference value of the LA deformation markers obtained by 3DSTE.

## Supplementary Information

Supplementary Information.

Supplementary Table S1.

Supplementary Table S2.

## Data Availability

The data sets analyzed in the current study are available from the corresponding author on reasonable request.
