# Supplementary material for: A systematic review and meta-analysis of the normal reference value of the longitudinal left atrial strain by three dimensional speckle tracking echocardiography
Source: Sci Rep. 2022 Mar 15;12:4395. doi: 10.1038/s41598-022-08379-7 (PMC8924244; doi:10.1038/s41598-022-08379-7)
Supplement: Supplementary file 1 — Supplementary Information. [file 41598_2022_8379_MOESM1_ESM.docx]

**Supplement**:

**PubMed**

("Left atrial" OR "left atrium") AND (strain OR speckle OR deformation) AND ("three-dimensional" OR "3-dimensional" OR "three dimensional" OR “3 dimensional” OR "three-dimension" OR "3-dimension" OR "three dimension" OR "3 dimension" OR "3D" OR "3-D")

Filter: the English Language

PubMed: 390

**Scopus**

(TITLE-ABS-KEY ("Left atrial" OR "left atrium") AND (strain OR speckle OR deformation) AND ("three-dimensional" OR "3-dimensional" OR "three dimensional" OR “3 dimensional” OR "three-dimension" OR "3-dimension" OR "three dimension" OR "3 dimension" OR "3D" OR "3-D") AND TITLE-ABS-KEY (strain OR speckle OR deformation) AND TITLE-ABS-KEY ("Left atrial" OR "left atrium" ) )

Filter: the English Language

Scopus: 269

**Embase**

Quick search:

("Left atrial" OR "left atrium") AND (strain OR speckle OR deformation) AND ("three-dimensional" OR "3-dimensional" OR "three dimensional" OR “3 dimensional” OR "three-dimension" OR "3-dimension" OR "three dimension" OR "3 dimension" OR "3D" OR "3-D")

Embase: 539
