# Supplementary material for: A systematic review and meta-analysis of the normal reference value of the longitudinal left atrial strain by three dimensional speckle tracking echocardiography
Source: Sci Rep. 2022 Mar 15;12:4395. doi: 10.1038/s41598-022-08379-7 (PMC8924244; doi:10.1038/s41598-022-08379-7)
Supplement: Supplementary file 2 — Supplementary Table S1. [file 41598_2022_8379_MOESM2_ESM.docx]

**Table 1S**: Full-text exclusion reasons

| First Author | Year of Publication | Exclusion Reasons |
| --- | --- | --- |
| Oliveira | 2008 | No 3DSTE data |
| Shin | 2009 | No 3DSTE data |
| Kleijn | 2011 | No 3DSTE data |
| Aktürk | 2012 | No 3DSTE data |
| Russo | 2012 | No 3DSTE data |
| Chadaide | 2013 | Repeated data set |
| Nascimento | 2013 | No 3DSTE data |
| Domsik | 2014 | Repeated data set |
| Hatipoglu | 2014 | No 3DSTE data |
| Kobayashi | 2014 | No healthy control group |
| Nemes | 2014 | No 3DSTE data |
| Onishi | 2014 | No 3DSTE data |
| Ren | 2014 | No 3DSTE data |
| Tadic | 2014 | No 3DSTE data |
| Tadic | 2014 | No 3DSTE data |
| Hayashi | 2015 | No 3DSTE data |
| Nemes | 2015 | Repeated data set |
| Ataş | 2016 | No 3DSTE data |
| Furukawa | 2016 | No healthy control group |
| Nemes | 2016 | No details of 3DSTE data (only presented in the figure), Repeated data set |
| Nemes | 2016 | Healthy control was not exactly screened |
| Nemes | 2016 | Repeated data set |
| Piras | 2016 | No 3DSTE data |
| Strachinaru | 2016 | No 3DSTE data |
| Tadic | 2016 | No 3DSTE data |
| Acıkgoz | 2017 | No 3DSTE data |
| Földeák | 2017 | Repeated data set |
| Havasi | 2017 | Repeated data set |
| Liao | 2017 | No 3DSTE data |
| Nemes | 2017 | Healthy control was not exactly screened |
| Russo | 2017 | No 3DSTE data |
| Schaaf | 2017 | No healthy control group |
| Braunauer | 2018 | No 3DSTE data |
| Genovese | 2018 | No 3DSTE data |
| Guedes | 2018 | Cardiovascular risk factors were not excluded |
| Kormányos | 2018 | No details of 3DSTE data (only presented in a figure), Repeated data set |
| Mohty | 2018 | Healthy control was not exactly screened |
| Mincu | 2018 | No 3DSTE data |
| Sugimoto | 2018 | No 3DSTE data |
| Vasconcellos | 2018 | No healthy control group |
| Zhao | 2018 | No 3DSTE data |
| Nemes | 2019 | No 3DSTE data |
| Nemes | 2019 | Global results only presented in a figure |
| Nemes | 2019 | No details of 3DSTE data |
| Saraiva | 2019 | No 3DSTE data |
| Takeuchi | 2019 | No 3DSTE data |
| Vasquez-Ortiz | 2019 | No 3DSTE data |
| Doria de Vasconcellos | 2020 | No healthy control group |
| Dogdus | 2020 | No healthy control group |
| Esposito | 2020 | No 3DSTE data |
| Lakatos | 2020 | Some subjects were less than 18 years old |
| Loncaric | 2020 | No 3DSTE data |
| Nemes | 2020 | Repeated data set |
| Parsaee | 2020 | No 3DSTE data |
| Saraiva | 2020 | No 3DSTE data |
| Sade | 2020 | No 3DSTE data |
| Dogdus | 2021 | No healthy control group |
| Dogdus | 2021 | No healthy control group |
| Jiang | 2021 | No 3DSTE data |
| Nemes | 2021 | No 3DSTE data |
| Zhu | 2021 | No 3DSTE data |
| Nemes | 2021 | No details of 3DSTE data (only presented in a figure) |
| Wan | 2021 | No 3DSTE data |

3DSTE: Three-dimensional speckle tracking echocardiography
