# Supplementary material for: A systematic review and meta-analysis of the normal reference value of the longitudinal left atrial strain by three dimensional speckle tracking echocardiography
Source: Sci Rep. 2022 Mar 15;12:4395. doi: 10.1038/s41598-022-08379-7 (PMC8924244; doi:10.1038/s41598-022-08379-7)
Supplement: Supplementary file 3 — Supplementary Table S2. [file 41598_2022_8379_MOESM3_ESM.docx]

**Table 2S**: Quality evaluation of included article

| No | Study | Year | Objective defined | Outcome described | Characteristics of controls described | Confounders described | Main findings outlined | Heterogeneous population | Strain imaging protocol | Individuals generating data blinded to outcomes | Sonographers blinded to outcome | Was reproducibility analysis performed? | Patients/control subjects recruited over same time period |
| --- | --- | --- | --- | --- | --- | --- | --- | --- | --- | --- | --- | --- | --- |
| 1 | Mochizuki et al | 2013 | Yes | Yes | Yes | Yes | Yes | NS | Yes | Yes | No | Yes | No |
| 2 | Aly et al | 2014 | Yes | Yes | No | Yes | Yes | NS | Yes | No | No | Yes | NS |
| 3 | Piros et al | 2016 | Yes | Yes | No | Yes | Yes | NS | Yes | NA | NA | No | NS |
| 4 | Esposito et al | 2019 | Yes | Yes | Yes | Yes | Yes | NS | Yes | No | No | Yes | Yes |
| 5 | Nabeshima et al | 2021 | Yes | Yes | Yes | No | Yes | NS | Yes | No | No | Yes | No |

NS: Not specified
